# On proper branched coverings and a question of Vuorinen

**DOI:** 10.1112/blms.12565

**Published:** 2022-03-04

**Authors:** Aapo Kauranen, Rami Luisto, Ville Tengvall

**Affiliations:** ^1^ Department de Matemàtiques Universitat Autònoma de Barcelona Bellaterra (Barcelona) Spain; ^2^ Digital Workforce Services Ltd. Mechelininkatu 1a Helsinki Finland; ^3^ Department of Mathematics and Statistics University of Helsinki Helsink Finland; ^4^ Department of Mathematics and Statistics University of Jyväskylä Jyväskylä Finland

## Abstract

We study global injectivity of proper branched coverings from the open Euclidean n‐ball onto an open subset of the Euclidean n‐space in the case where the branch set is compact. In particular, we show that such mappings are homeomorphisms when n=3 or when the branch set is empty. This gives a positive answer to the corresponding cases of a question of Vuorinen.

## 1 INTRODUCTION

For a continuous, open, and discrete mapping $f:\Omega \to \Omega^{\prime }$ between Euclidean domains we define its *branch set*, denoted as $B_{f}$, to be the set of points where f is not a local homeomorphism. Notice that throughout the paper by *domain*, we mean a connected open subset of $R^{n}$. The structure of this set is tied to the topology and geometry of the mapping itself, but in general the structure of the branch set is not well understood. Even for the important special class of continuous, open, and discrete maps called quasiregular mappings, many properties of the branch set remain largely unknown, but the topic garners great interest. In his ICM address [16, Section 3], Heinonen wrote: “There is currently no theory available that would explain or describe the geometry of allowable branch sets.” This leads us to the following broad question:

Can we describe the geometry and the topology of the allowable branch sets of quasiregular mappings between metric n‐manifolds?

In this paper we focus on a particular aspect of this general problem known as *Vuorinen's question* concerning the compactness of the branch set of *proper* continuous, open, and discrete mappings. The question is as follows:

Suppose that $f:B^{n}\to f\left(B^{n}\right)\subset R^{n}$, n⩾3, is a proper, continuous, open, and discrete mapping with a compact branch set $B_{f}$. Is f then a homeomorphism?

In this paper we will refer to this question simply as *the Vuorinen question*. The question first appeared in the work of Vuorinen [33, Remarks 3.7] on the boundary behavior of quasiregular mappings. Later it was stated in the well‐known monograph [35, p. 193, (4)] and the query [34] of the same author. It was further promoted by Srebro in a survey collection [36, p. 108], and also given in the slightly stronger setting of quasiregular mappings in the collections [5, p. 503, 7.66] and [15, p. 180, Problem 7.66] of research problems in complex analysis. Our first main result gives a positive answer to the question in dimension three.

Theorem 1.1 Let $f:B^{3}\to f\left(B^{3}\right)\subset R^{3}$ be a proper, continuous, open, and discrete map. If $B_{f}$ is compact, then f is a homeomorphism.

The crucial idea of the proof is to investigate the existence of torsion elements of the fundamental group of the image of the underlying map. If such elements do not exist, then the mapping is a homeomorphism; for the precise statement see Proposition 4.3. We furthermore show that the claim is true in all dimensions when the branch set is empty.

Theorem 1.2 Let $f:B^{n}\to f\left(B^{n}\right)\subset R^{n}$ be a proper, continuous, open, and discrete map with n⩾2. If $B_{f}=\emptyset$, then f is a homeomorphism.

Theorem 1.2 follows easily from well‐known facts in algebraic topology. Indeed, first one observes that a proper local homeomorphism is a covering map, see Lemma 3.1 or [22, Exercise 11‐9]. Hence $f(B^{n})$ is a finite dimensional Eilenberg–MacLane space K(G,1) with a finite group $G=\pi_{1}\left(f\left(B^{n}\right)\right)$, see Lemma 3.3. Then by basic algebraic topology, see, for example, [14, Proposition 2.45, p. 149], one notices that this is possible only if the fundamental group of $f(B^{n})$ is trivial and f is therefore a homeomorphism. However, we have not been able to find any proof for Theorem 1.2 from the literature. Furthermore, the result or its proof is not well known for most experts in the field of geometric mapping theory who we expect to be the main audience for this research. Therefore, we have recorded it with a detailed proof in this article.

The results in the paper should be contrasted with Zorich's global homeomorphism theorem, see, for example, [37] or [29, Corollary III.3.8], which states that if n⩾3, then an entire quasiregular mapping $R^{n}\to R^{n}$ with an empty branch set is always quasiconformal, that is, a homeomorphic quasiregular mapping. In addition, the origin of Vuorinen's question is in the study of induced boundary mappings of closed quasiregular mappings, see [32, section 5] and especially [32, Theorem 5.3]. In this context our results can be used to study whether it is possible to produce extra branching to a quasiregular mapping by changing the mapping only locally. However, at least in three dimensions Theorem 1.1 prohibits the non‐global topological modifications of these mappings by providing a positive answer to the following question from [35, Open problem 9.18, p. 125]:

Let $f:\Omega \to R^{n}$ be a branched covering with $\Omega \subset R^{n}$ a domain. Suppose $x_{0}\in \Omega$ and $r\in (0,d\left(x_{0},\partial \Omega \right))$. If $B(x_{0},r)$ is a normal domain of f and $B_{f}\cap \partial B\left(x_{0},r\right)=\emptyset$, is ${f|}_{B(x_{0},r)}$ then necessarily injective?

In our proof of Theorem 1.1, a crucial step is to show that in all dimensions the claim follows whenever the image has torsion‐free fundamental group at infinity, see Definition 4.2 and Proposition 4.3. The Vuorinen question in three dimensions is then answered after noting that any domain in $R^{3}$ has a torsion‐free fundamental group by known results in topology. In contrast, in higher dimensions the fundamental group of a Euclidean domain can have torsion elements, as is exemplified, for example, with a tubular neighborhood of a real projective plane embedded in $R^{4}$, and so our proof does not generalize to all dimensions. Thus, the general case of the Vuorinen question is still open in dimensions four and above. For the case when the branch set is empty, Theorem 1.2, the proof also relies on the study of the existence of torsion elements in the fundamental group of the image and we rely on the theory of K(G,1)‐spaces; see Section 3.

We note that the proof of Theorem 1.1 actually gives rise to the following result.

Proposition 1.3 Let $f:M\to f\left(M\right)\subset R^{3}$ be a proper branched covering where M is an open 3‐manifold simply connected at infinity. Suppose that $B_{f}$ is compact. Then f is a homeomorphism.

Likewise we obtain the following more general version of Theorem 1.2.

Proposition 1.4 Let $f:M\to f\left(M\right)\subset R^{n}$ be a proper branched covering where M is an open n‐manifold with n⩾2. If $M\setminus f^{-1}\left(f\left(B_{f}\right)\right)$ has a contractible universal cover, then f is a homeomorphism.

In the more restricted setting of quasiregular mappings between hyperbolic manifolds, related questions have been studied by Bridson, Hinkkanen, and Martin in [8].

Note that the answer to the question of Vuorinen is negative in dimension two unless the branch set is assumed to be empty as is demonstrated by the planar winding map. However, in higher dimensions the winding map does not serve as a counterexample as the branch set of this map is an (n−2)‐dimensional plane and thus not compact. Non‐empty compact branch sets are also possible to construct for continuous, open, and discrete mappings $B^{n}\to R^{n}$, see, for example, [21], but the known examples are no longer proper maps. In addition, non‐injective local homeomorphisms $B^{n}\to R^{n}$ can be exemplified with the mapping

(1.1) $$\begin{array}{c} \psi :{\left(0,4\pi \right)}^{2}\times \left(0,1\right)\to R^{3},\;\psi \left(z,t\right)\mapsto \left(\exp\left(z\right),t\right) \end{array}$$

and its higher dimensional analogs, but this mapping fails to be proper as well. Note that all the above‐mentioned mappings are quasiregular mappings as well.

The examples in the preceding paragraph seem to hint that the challenge in the solution, or a possible counterexample, to the Vuorinen question might lie in trying to balance the properness of the mapping with the compactness of the branch set. Furthermore we note that, as Proposition 4.3 demonstrates, a possible counterexample to the Vuorinen question must display some nontrivial structure as the image of the map must have a complicated boundary, in some sense. We also remark that the branch set itself can also exhibit very complicated structure. Indeed, for a continuous, open and discrete mapping whose branch set image is topologically piecewise linear, the mapping is itself locally equivalent to a combination of winding maps; see, for example, [24]. On the other hand, there are mappings which do not exhibit such simple behavior, notably the Heinonen–Rickman map whose branch set contains a wild Cantor set ([19]) and the classical example of Church and Timourian from [11] which is based on deep work of Cannon and Edwards, see, for example, [9] and the references within. For further discussion, see [2, 24].

## 2 PRELIMINARIES

### 2.1 Notation

Throughout this paper we denote by Ω a domain in n‐dimensional Euclidean space $R^{n}$ with n⩾2. A point $x\in R^{n}$ in coordinates is denoted by $x=(x_{1},x_{2},\text{…},x_{n})$, and its Euclidean norm is denoted by $|x|≔\sqrt{\sum_{i=1}^{n}x_{i}^{2}}$. An n‐dimensional (open) ball in $R^{n}$ of radius r>0, centered at $a\in R^{n}$, is denoted by

$$B^{n}\left(a,r\right)≔\{x\in R^{n}:|x-a|<r\},$$

and if the ball is centered at the origin, we sometimes denote it by $B_{r}^{n}$ or by $B^{n}$ when r=1. If the dimension of the ball does not play a role, we may exclude it from the notation. Moreover, if we want to emphasize that a ball B(a,r) needs to be considered as a ball of some metric space Y, we may denote $B_{Y}\left(a,r\right)$. The topological interior of a set $A\subset R^{n}$ will be denoted as $A^{\circ }$ and the closure by $\bar{A}$. The topological boundary of a set A is denoted by ∂A. The number of points in a set $A\subset R^{n}$ is denoted by

#A≔card(A).

A continuous mapping $\gamma :I\to R^{n}$ of an interval I⊂R is called *path* and its image is denoted by $\left|\gamma |≔\{y\in R^{n}:y=\gamma \left(t\right)\;\text{for}\;\text{some}\;t\in I\right\}$.

### 2.2 Branched coverings and related mapping classes

A mapping f:X→Y between metric spaces is said to be

(i) *open* if it maps every open set in X to an open set in Y,
(ii) *discrete* if the set of preimages is a discrete set in X for every point in Y,
(iii) *proper* if the preimage of every compact set in Y is a compact set in X,
(iv) *a branched covering map*, or more informally *a branched covering*, if it is continuous, discrete, and open,
(v) a *local homeomorphism at a point* x∈X if there is an open neighborhood V⊂X of x such that the restriction ${f|}_{V}:V\to f\left(V\right)\subset Y$ of f is a homeomorphism,
(vi) a *local homeomorphism* if it is a local homeomorphism at every point x∈X, and
(vii) a *covering map* if it is continuous surjection such that each point y∈Y has an open neighborhood V such that $f^{-1}\left(V\right)$ is a disjoint union of open sets, each of which is mapped homeomorphically by f onto V.

Note that even though the definitions (i)–(vii) above are given for mappings between metric spaces, in what follows we consider mainly mappings

f:U→f(U),

where both spaces X=U and Y=f(U) are subsets of $R^{n}$ endowed with the usual Euclidean metric.

Note that every branched covering $f:U\to R^{n}$ is a local homeomorphism outside its *branch set*

$$\begin{array}{c} B_{f}≔\left\{x\in U:f\;\text{is}\;\text{not}\;a\;\text{local}\;\text{homeomorphism}\;\text{at}\;x\right\}. \end{array}$$

The most elementary example of a proper branched cover that is not a local homeomorphism is the m *‐to‐1 winding mapping* $w_{m}:R^{n}\to R^{n}$ defined in cylindrical coordinates by the formula

$$\begin{array}{c} w_{m}\left(r,\theta ,x_{3},\text{…},x_{n}\right)=\left(r,m\theta ,x_{3},\text{…},x_{n}\right), \end{array}$$

with some given integer m such that |m|⩾2. The study of continuous, open, and discrete mappings has a solid history which can be studied more, for instance, from [2, 6, 10, 27] and the references therein.

An important subclass of branched coverings is the class of quasiregular mappings. A mapping $f:\Omega \to R^{n}$ is called K *‐quasiregular* with K⩾1 if

(i) it belongs to Sobolev space $W_{\text{loc}}^{1,n}\left(\Omega ,R^{n}\right)$, and
(ii) it satisfies the *distortion inequality*
$${∥Df\left(x\right)∥}^{n}⩽KJ_{f}\left(x\right)$$
  for almost every x∈Ω.

Above ∥Df(x)∥ refers to the operator norm of the weak differential matrix Df(x) at a point x∈Ω, and $J_{f}\left(x\right)≔\det Df\left(x\right)$ stands for the Jacobian determinant of f at a point x∈Ω.

For the basic knowledge on quasiregular mappings, we refer to [29, 35]. By the Reshetnyak theorem quasiregular mappings are branched coverings ([28] or [29, Section IV.5, p. 145]), and so, branched coverings can be seen as generalizations of quasiregular mappings, see, for example, [23]. For further discussion on quasiregular mappings and other related mapping classes, we refer to [4, 17 20, 25 31].

The term *branched cover(ing)* is widely used in the theory of quasiregular mappings to mean continuous, open, and discrete mappings. However, the term is not standard even in closely related fields, and thus, we will explore the nomenclature a bit. In particular, we wish to comment on how a branched covering relates to *covering maps*.

For *proper* branched coverings the connection to covering maps is quite immediate. Indeed, when a surjective branched covering f:X→Y between locally compact and complete path‐metric spaces is assumed to be proper, then it is actually a covering map when restricted to the set

$$\begin{array}{c} X\setminus f^{-1}\left(f\left(B_{f}\right)\right). \end{array}$$

In Lemma 3.1 this is shown in the case $B_{f}=\emptyset$. The general case follows from the simple observation that for a proper branched covering f:X→Y also the restriction $f:f^{-1}\left(V\right)\to V$ is a proper branched cover for any open set V. Note, however, that in general the restriction of a branched covering f:X→Y to the complement of $f^{-1}\left(f\left(B_{f}\right)\right)$ does not yield a covering map; see, for example, [1] for some further discussion.

From this point of view we note that in [7] branched coverings are defined to be locally equivalent to winding maps, in [6] the mappings are studied only between closed manifolds which implies properness, in [13] a branched cover f:X→Y is a map that is a “completion” of a covering map defined on an open dense subset of X, and in [27] branched covers are only studied in the PL‐category where properness also follows. This list should not be considered to be in any way exhaustive, but does demonstrate that the term branched covering needs to be used carefully. In our setting a branched covering needs not to be proper, but we do note that every point in the domain always has a neighborhood basis of normal domains U with the property that the restriction of f to U is proper. By further restiricting to the set

$$U\setminus f^{-1}\left(f\left(B_{f}\cap U\right)\right)$$

and by applying Lemma 3.1 we actually obtain a covering map as explained in the previous paragraph.

### 2.3 Normal domains and path‐lifting

We follow the conventions of [29] and say that a domain U⊂X is a *normal domain* for a branched covering f:X→Y if U is compactly contained in X and

$$\begin{array}{c} \partial f(U)=f(\partial U). \end{array}$$

A normal domain U is *a normal neighborhood* of x∈U if

$$\begin{array}{c} \bar{U}\cap f^{-1}\left(f\left(x\right)\right)=\left\{x\right\}. \end{array}$$

If Y is a metric space, then we denote by U(x,f,r) the component of the open set $f^{-1}\left(B_{Y}\left(f\left(x\right),r\right)\right)$ containing x. The existence of arbitrarily small normal neighborhoods is essential for the theory of branched covers. The following lemma guarantees the existence of normal domains, and the proof can be found in [29, Lemma I.4.9, p. 19] (see also [30, Lemma 5.1.]).

Lemma 2.1 Let X and Y be locally compact complete path‐metric spaces and f:X→Y a branched cover. Then for every point x∈X there exists a radius $r_{0}>0$ such that U(x,f,r) is a normal neighborhood of x for any $r\in (0,r_{0})$. Furthermore,

$$\begin{array}{c} \lim_{r\to 0}\mathrm{diam}U\left(x,f,r\right)=0. \end{array}$$

Finally, a fundamental technique in the study of branched covers is the path‐lifting. For the terminology and basic theory of this technique, we refer to [29, Section 3, p. 32].†

Lemma 2.2 Let $\Omega \subset R^{n}$, n⩾2, be a domain and suppose $f:\Omega \to f\left(\Omega \right)\subset R^{n}$ is a proper branched cover. Then for any path β:[0,1]→f(Ω) and any $x\in \Omega \cap f^{-1}\left(\beta \left(0\right)\right)$ there exists a path α:[0,1]→Ω for which f∘α=β and α(0)=x. Such a path is called a lift of β *(*under f *)*.

By [29, Corollary *3.3*, p. 34] there exists a maximal lift γ:I→Ω of β such that γ(0)=x, where I is a subinterval of [0,1] of type [0,t] or [0,t) for some t∈(0,1]. We need to show that I=[0,1]. Toward contradiction suppose not, and assume first that I is a closed interval I=[0,a], a>0. But now since a<1 we may again take by [29, Corollary *3.3*., p. 34] a maximal lift of ${\beta |}_{\left[a,1\right]}$ starting from the point γ(a) and concatenate this lift to γ. This contradicts the maximality of the lift γ, and we deduce that I must be open, that is, of the form [0,b). We wish to show next that in this case the limit $\lim_{t\to b}\gamma \left(t\right)$ exists.

Note first that since f is a discrete mapping, the set $f^{-1}\left(\beta \left(b\right)\right)$ is a discrete subset of Ω. Furthermore since f is a proper map, the pre‐image of the singleton β(b) must be a compact set. As a compact and discrete subset of the Euclidean space, it is thus a finite set. Now we can choose $\varepsilon_{0}>0$ in such a way that the closed balls $\bar{B}\left(x_{j},\varepsilon_{0}\right)$, $x_{j}\in f^{-1}\left(\beta \left(b\right)\right)$, are disjoint and compactly contained in Ω. Since f is an open mapping, the images of the corresponding open balls $B(x_{j},\varepsilon_{0})$, $x_{j}\in f^{-1}\left(\beta \left(b\right)\right)$ are open sets in f(Ω), all containing β(b). Thus, by Lemma 2.1, there exists a radius $r_{0}>0$ such that for each $x_{j}\in f^{-1}\left(\beta \left(b\right)\right)$ the set $U(x_{j},f,r_{0})$ is a normal domain of $x_{j}$ with $U\left(x_{j},f,r_{0}\right)\subset B\left(x_{j},\varepsilon_{0}\right)$. Now we note that since β is continuous, there exists a $\delta_{0}>0$ such that for any $t\in (b-\delta_{0},b)$, $\beta \left(t\right)\in B(\beta \left(b\right),r_{0})$. In particular, the subpath ${\gamma |}_{\left(b-\delta ,b\right)}$ must be contained in the union of the finitely many normal domains $U(x_{j},f,r_{0})$, $x_{j}\in f^{-1}\left(\beta \left(b\right)\right)$. Since this subpath is a connected set, it must be contained in one of these disjoint normal domains, say $U(x_{j_{0}},f,r_{0})$, and thus in the ball $B(x_{j_{0}},\varepsilon_{0})$. Now by repeating the argument above for any $\varepsilon \in (0,\varepsilon_{0})$, we see that for any such ε there exists a δ>0 such that for all t∈(b−δ,b) we have $\gamma \left(t\right)\in B\left(x_{j_{0}},\varepsilon \right)$, and thus $\lim_{t\to b}\gamma \left(t\right)=x_{j_{0}}$. This implies that the lift γ can be extended to the closed interval [0,b] since by the continuity of f we have f(γ(b))=β(b). This is again a contradiction with the maximality of γ, and so the original claim holds true; I=[0,1] and we may choose α=γ.□

## 3 PROOF OF THEOREM 1.2

The proof in the setting of no branch set relies on the fact that the mapping $f:B^{n}\to f\left(B^{n}\right)\subset R^{n}$ is in fact a covering map defined on a contractible n‐manifold. This observation can be used to show that the image $f(B^{n})$ is actually *an Eilenberg–MacLane space* K(G,1), that is, a path‐connected space whose fundamental group is isomorphic to a group G and which has contractible universal covering space, see, for instance, [14, p. 87 onward].

After this we use the notion of Eilenberg–MacLane spaces to rule out the examples with fundamental groups that are not torsion‐free which could potentially arise in higher dimensions. We start by showing that a proper local homeomorphisms between metric spaces are a covering maps. For further results on the topic, see, for example, [18].

Lemma 3.1 A map f:X→Y between metric spaces which is proper and a local homeomorphism is a covering map.

Fix a point y∈Y. Since f is proper and discrete it follows that $f^{-1}\left(y\right)$ is a compact and discrete set. Hence $f^{-1}\left(y\right)$ is finite and we may write

$$f^{-1}\left(y\right)=\left\{x_{1},x_{2},\text{…},x_{m}\right\}$$

for some distinct points $x_{1},x_{2}\text{…},x_{m}$ in X.

Next, because f is a local homeomorphism, we may consider pairwise disjoint $x_{i}$‐centric open balls

$$B_{i}≔B\left(x_{i},r_{i}\right)\subset X\;\;(i=1,\text{…},m)$$

with positive radii such that the restrictions

$$f_{i}{=f|}_{B_{i}}:B_{i}\to f\left(B_{i}\right)\;\;(i=1,\text{…},m)$$

are homeomorphisms. Set

$$V=\cap_{i=1}^{m}f\left(B_{i}\right).$$

Then by the openness of f and the way we have chosen the balls $B_{i}$, it follows that V is open and it contains the point y.

Next we set

$$U_{i}≔B_{i}\cap f^{-1}\left(V\right)\;.$$

We claim that there exists a ball $\hat{B}\subset V$ centered at y such that

$$f^{-1}\left(\hat{B}\right)=U_{1}^{\prime }\cup \cdots \cup U_{m}^{\prime }$$

for $U_{i}^{\prime }=U_{i}\cap f^{-1}\left(\hat{B}\right)$. If this would not be the case, there would be a shrinking sequence of open balls ${\hat{B}}_{k}≔B\left(y,{\hat{r}}_{k}\right)$ centered at y, and points $z_{k}\in f^{-1}\left({\hat{B}}_{k}\right)$ not contained in any $U_{i}$ such that the sequence $f(z_{k})$ is converging to y. The set

$$K=\{y,f\left(z_{1}\right),f\left(z_{2}\right),\text{…}\}$$

is compact and hence by the properness of f also its preimage $f^{-1}\left(K\right)$ is compact. This implies that the sequence $z_{k}$ has a convergent subsequence converging to a point in $f^{-1}\left(y\right)$, say to the point $x_{i}$. However, this is impossible since all the points $z_{k}$ lie outside the set $U_{i}$. Thus there exists an open ball $\hat{B}$ centered at y such that

$$f^{-1}\left(\hat{B}\right)=U_{1}^{\prime }\cup \cdots \cup U_{m}^{\prime }$$

where $U_{i}^{\prime }$ are open sets in X mapped homeomorphically onto $\hat{B}$ by f. This shows that f is a covering map.□

The required torsion‐freeness property is well known in the literature. The following statement can be found in [14, Proposition 2.45, p. 149].

Proposition 3.2 Let Y be a finite‐dimensional CW‐complex. If Y is a K(G,1)‐space, then $G=\pi_{1}\left(Y\right)$ is torsion‐free.

The advantage of Proposition 3.2 is that it can be used to provide the torsion‐freeness of the fundamental group of the target without any additional assumption on the dimension. Whenever this property of the fundamental group of the target is verified, we can give a positive answer to Vuorinen question with the techniques introduced in this paper. Besides Proposition 3.2 we need also the following simple lemma (Lemma 3.3) to prove Theorem 1.2. We note that Lemma 3.3 is known to the experts in the field, but we have not seen it explicitly stated in the literature, so we provide a proof for the convenience of the reader.

Lemma 3.3 Let f:X→Y is a proper covering map between path‐connected metric spaces. If X is simply connected, then $\pi_{1}\left(Y\right)$ is finite.

Take a point y∈Y. Since f is proper and discrete the set $f^{-1}\left(y\right)=\left\{x_{1}\text{…},x_{m}\right\}$ is finite. For each point $x_{i}\in f^{-1}\left(y\right)$ choose a path $\gamma_{i}:\left[0,1\right]\to X$ from $x_{1}$ to $x_{i}$. With the help of these paths define a function

$$\Psi :f^{-1}\left(y\right)\to \pi_{1}\left(Y\right),\;\Psi \left(x_{i}\right)=\left[f\circ \gamma_{i}\right]\;,$$

where $\left[f\circ \gamma_{i}\right]$ denotes the homotopy class of the loop $f\circ \gamma_{i}$ in Y. Since X is simply connected, any two paths joining $x_{1}$ and $x_{i}$ are homotopic and thus their images under f are also homotopic. This shows that the function Ψ is well defined. The function Ψ is also surjective. To see this, notice that for any loop γ in Y that starts and ends at y lifts to a path ${\tilde{\gamma }}_{i}$ that joins $x_{1}$ to some $x_{i}\in f^{-1}\left(y\right)$. Thus it follows that

$$\Psi \left(x_{i}\right)=\left[f\circ \gamma_{i}\right]=\left[f\circ {\tilde{\gamma }}_{i}\right]=\left[\gamma \right]\;,$$

which gives the surjectivity of Ψ. Therefore

$$\#\pi_{1}\left(Y\right)⩽\#f^{-1}\left(y\right)<\infty ,$$

which ends the proof.□

Proof of Theorem 1.2 Suppose that $f:B^{n}\to f\left(B^{n}\right)\subset R^{n}$ is a proper branched covering such that $B_{f}=\emptyset$. Denote

$$Y≔f\left(B^{n}\right)\subset R^{n}\;\text{and}\;G≔\pi_{1}\left(Y\right).$$

By Lemma 3.1 f is a covering map, and since $B^{n}$ is simply connected, it is the universal cover of Y. This means, by definition, that Y is an Eilenberg–MacLane space K(G,1) for $G=\pi_{1}\left(Y\right)$, see [14, p. 87].

Next, since Y is an open set in $R^{n}$, it can be given the structure of a CW‐complex. Thus, by Proposition 3.2 the fundamental group $G=\pi_{1}\left(Y\right)$ has no torsion. Furthermore, by Lemma 3.3 we then note that $\pi_{1}\left(Y\right)$ must be finite. Therefore, as a finite torsion‐free group $\pi_{1}\left(Y\right)$ is trivial. We will use this observation to show that f injective.

In order to see that f is injective take $y\in f(B^{n})$ and fix any two points $x_{1},\;x_{2}\in f^{-1}\left(y\right)$. Let γ be a path joining $x_{1}$ to $x_{2}$ in $B^{n}$. Since $\pi_{1}\left(Y\right)$ is trivial, we know that f∘γ is equivalent to the constant path with a homotopy that keeps the endpoints of the loop at y at all times during the homotopy. This homotopy lifts to a homotopy $\gamma_{t}$, t∈[0,1] of the path $\gamma =\gamma_{0}$ that keeps the endpoints fixed at $x_{1}$ and $x_{2}$. On the other hand $\gamma_{1}$ is a constant path as a lift of a constant path. Therefore we have $x_{1}=x_{2}$ and thus f is injective. Especially, $f:B^{n}\to Y$ is then a global homeomorphism as a continuous and open bijection.□

## 4 PROOF OF THEOREM 1.1

We start by proving a lemma which provides a useful collection of large normal domains in the setting of the Vuorinen question. In what follows a connected component $\hat{E}$ of a set E⊂Ω is called a *boundary component of* E if its closure in Ω is not compact. Note that a set E can have several boundary components.

Lemma 4.1 Let

$$f:\Omega \to f\left(\Omega \right)\subset R^{n}\;\left(\Omega \subset R^{n}\;\text{domain}\;\text{with}\;n⩾2\right)$$

be a proper branched covering and let K⊂f(Ω) be a non‐empty compact set. Denote

$$C≔f^{-1}\left(K\right)\subset \Omega$$

and suppose that V⊂Ω is a domain such that $C\subset V\subset \bar{V}\subset \Omega$. Then for the set $U≔f^{-1}\left(f\left(V\right)\right)$ we have the following:

(a) U is a path‐connected open set such that f(U)=f(V),
(b) U is a normal domain for f,
(c) ${f|}_{U}:U\to f\left(U\right)$ is a proper branched cover,
(d) ${f|}_{\Omega \setminus \bar{U}}:\Omega \setminus \bar{U}\to f\left(\Omega \right)\setminus f\left(\bar{U}\right)$ is a proper branched cover and $\Omega \setminus \bar{U}=f^{-1}\left(f\left(\Omega \setminus \bar{U}\right)\right)$, and
(e) if a set E⊂Ω is a boundary component of $\Omega \setminus \bar{U}$, then f(E) is a boundary component of $f\left(\Omega \right)\setminus f\left(\bar{U}\right)$. Moreover, if a point y is contained in a boundary component of $f\left(\Omega \right)\setminus f\left(\bar{U}\right)$, then all its preimages are contained in boundary components of $\Omega \setminus \bar{U}$.

Note that since V is a domain and f an open map, f(V) is open and so is its preimage U under the continuous map f.

(a) For the second claim of (a) we simply note that
$$f\left(U\right)=f(f^{-1}\left(f\left(V\right)\right))=f\left(V\right).$$
  For the path‐connectedness of U we first note that f(V) is a domain containing the compact set K. Therefore, for any point x∈U we may connect f(x) and K with a path α:[0,1]→f(V). By Lemma 2.2 the path α has a lift $\tilde{\alpha }:\left[0,1\right]\to \Omega$ with $\tilde{\alpha }\left(0\right)=x$ and by the definition of U we have $|\tilde{\alpha }|\subset U$. On the other hand $\tilde{\alpha }\left(1\right)\in C\subset V$, and so each point x∈U can be connected with a path to an interior point of the connected set V⊂U. This implies that U is path‐connected.
(b) By (a) it is enough to show that ∂f(U)=f(∂U). Openness of f gives the inclusion
∂f(U)⊂f(∂U).
  For the second inclusion fix a point y∈f(∂U). If $U\cap f^{-1}\left(y\right)\neq \emptyset$, then U is a neighborhood of one of the preimages of y. Then by the openness of f we see that f(U)=f(V) is a neighborhood of y. This implies that $U=f^{-1}\left(f\left(V\right)\right)$ is a neighborhood of all the points in the preimage of y. Therefore, we have
$$\partial U\cap f^{-1}\left(y\right)=\emptyset ,$$
  which is a contradiction as y∈f(∂U). Thus, we have proved that $U\cap f^{-1}\left(y\right)=\emptyset$ and so y∈∂f(U) since $y\in \bar{f(U)}$. This gives us
∂f(U)⊃f(∂U).
(c) The set U is a domain by (a) and the restriction of a branched covering to a domain is a branched covering. To show that ${f|}_{U}:U\to f\left(U\right)$ is proper, we fix a compact set A⊂f(U) and note that $f^{-1}\left(A\right)\subset \Omega$ is compact since f is proper. Now as $U=f^{-1}\left(f\left(U\right)\right)$, we have that $f^{-1}\left(A\right)\subset U$, and so we see that ${\left(f{|}_{U}\right)}^{-1}\left(A\right)$ is compact. Thus ${f|}_{U}$ is proper.
(d) First we note that the restriction of a branched covering to an open set is a branched covering. Since by part (b) we have ∂f(U)=f(∂U), we see that also
(4.1) $$\begin{array}{c} f\left(\partial \left(\Omega \setminus \bar{U}\right)\right)=\partial f\left(\Omega \setminus \bar{U}\right), \end{array}$$
  where the boundary is taken relative to the domain f(Ω). As in part (c), the properness will follow after we show that
$$\begin{array}{c} \Omega \setminus \bar{U}=f^{-1}\left(f\left(\Omega \setminus \bar{U}\right)\right). \end{array}$$
  The inclusion
$$\begin{array}{c} \Omega \setminus \bar{U}\subset f^{-1}\left(f\left(\Omega \setminus \bar{U}\right)\right) \end{array}$$
  is trivial, so fix a point $x\in f^{-1}\left(f\left(\Omega \setminus \bar{U}\right)\right)$. Suppose, toward a contradiction, that $x\notin \Omega \setminus \bar{U}$. Then either x∈∂U or x∈U. In the first case we have by applying (4.1) that $f\left(x\right)\in \partial f\left(\Omega \setminus \bar{U}\right)$, which is not possible because by the choice of x we have $f\left(x\right)\in f\left(\Omega \setminus \bar{U}\right)$. In the second case we get by the definition of U that $f^{-1}\left(f\left(x\right)\right)\subset U$. Thus, we have $x\notin \Omega \setminus \bar{U}$ which again goes against our assumptions. Therefore, we conclude that $x\in \Omega \setminus \bar{U}$ and so
$$\begin{array}{c} \Omega \setminus \bar{U}⊃f^{-1}\left(f\left(\Omega \setminus \bar{U}\right)\right), \end{array}$$
  which ends the proof of the claim.
(e) Let E be first some *(*not necessarily a boundary*)* component of $\Omega \setminus \bar{U}$. First we show that E is mapped onto some component of $f\left(\Omega \right)\setminus f\left(\bar{U}\right)$. It is clear that f(E) is contained in some component $C_{1}$ of $f\left(\Omega \right)\setminus f\left(\bar{U}\right)$. If $C_{1}\neq f\left(E\right)$, we find a point $y\in C_{1}\setminus f\left(E\right)$ and a sequence ${\left\{y_{j}\right\}}_{j\in N}$ in f(E) converging to y. Notice that by the definition of U we have $f^{-1}\left(y\right)\subset \Omega \setminus \bar{U}$. Choose a sequence ${\left\{x_{j}\right\}}_{j\in N}$ in E such that $f\left(x_{j}\right)=y_{j}$. Since $f^{-1}\left(\left\{y,y_{1},y_{2},\text{…}\right\}\right)$ is compact, we see that $\left\{x_{j}\right\}$ has a convergent subsequence *(*also denoted by $x_{j}$ *)*, which converges to a point x. By continuity we see that $x\in f^{-1}\left(y\right)$ and thus x∉E but since $y\notin f(\bar{U})$ we have $x\in \Omega \setminus \bar{U}$. Thus x is in a different component than all $x_{j}$. This is a contradiction and therefore every component of $\Omega \setminus \bar{U}$ is mapped onto some component of $f\left(\Omega \right)\setminus f\left(\bar{U}\right)$ by the mapping f.
  If E is a boundary component of $\Omega \setminus \bar{U}$ and its image is not a boundary component of $f\left(\Omega \right)\setminus f\left(\bar{U}\right)$, then since f is proper, $f^{-1}\left(f\left(E\right)\right)$ would have a compact closure which is not possible as E is a boundary component. This proves the first claim. Continuity now implies that other components are not mapped to boundary components, which implies the second claim.□

The proof of Theorem 1.1 relies on deep results in low‐dimensional topology, namely, Proposition 4.4. For the statement of the result we need some auxiliary concepts. We refer to [14] for the definition and the basic properties of the fundamental group $\pi_{1}\left(X\right)$ of a space X.

Definition 4.2 We say that a domain $\Omega \subset R^{n}$ has *torsion‐free fundamental group at infinity* if for any compact set K⊂Ω there exists a domain V⊃K with $\bar{V}$ being compact in Ω and such that $\pi_{1}\left(\Omega \setminus \bar{V}\right)$ is torsion‐free; recall that a group is torsion‐free if no element g≠e has the property that $g^{j}=e$ for some j∈N, where e is the neutral element of the group.

The nomenclature of this definition is motivated by a similar definition of a space being *simply connected at infinity*, see, for example, [12].

The following proposition is the fundamental observation in the proof of our first main theorem. We wish to emphasize that Proposition 4.3 is valid in all dimensions n⩾3.

Proposition 4.3 Let $f:B^{n}\to f\left(B^{n}\right)\subset R^{n}$ be a proper branched covering with n⩾3. Suppose that $f(B^{n})$ has torsion‐free fundamental group at infinity. If $B_{f}$ is compact, then f is a homeomorphism.

Since $B_{f}$ is compact and f is a continuous proper map, both $f(B_{f})$ and $f^{-1}\left(f\left(B_{f}\right)\right)$ are also compact. Thus there exists $r_{0}\in \left(0,1\right)$ such that $f^{-1}\left(f\left(B_{f}\right)\right)\subset B_{r_{0}}$. For any $r\in [r_{0},1)$, we denote

$$\begin{array}{c} U_{r}≔f^{-1}\left(f\left(B_{r}\right)\right),\;\;\text{and}\;\;E_{r}≔B\setminus {\bar{U}}_{r}. \end{array}$$

By Lemma 4.1 (c) and (d) the restriction of f to either one of these sets will be a proper branched cover.

**FIGURE 1:**
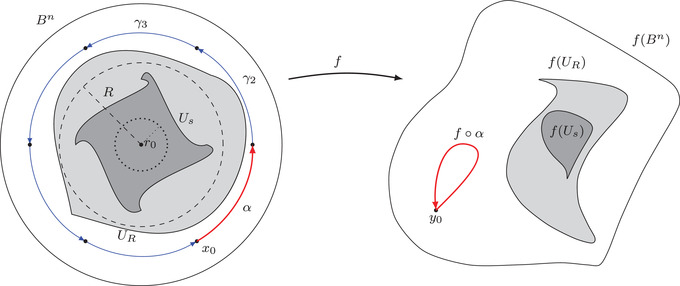
Essential objects in the proof of Proposition 4.3

Since we assumed f(B) to be torsion‐free at infinity, there exists a compact set K⊂f(B) such that $K⊃f({\bar{B}}_{r_{0}})$ and f(B)∖K have a torsion‐free fundamental group. We fix now a radius $s\in (r_{0},1)$ for which $K\subset f(B_{s})$, and take R∈(s,1) to be such that ${\bar{U}}_{s}\subset B_{R}$; see Figure *1*. Since $f:B\to R^{n}$ is a proper branched cover, we note that all points in f(B) have a finite number of preimages. In particular we note that since $B_{f}\subset B_{s}\subset B_{R}$, all the points in

$$\begin{array}{c} f\left(B\right)\setminus f\left({\bar{B}}_{R}\right)=f\left(E_{R}\right) \end{array}$$

have finite number of preimages in B. Now, by Lemma 4.1(d) and Lemma 3.1 we see that ${f|}_{E_{R}}:E_{R}\to f\left(E_{R}\right)$ is a covering map.

Let C be the unique boundary component of the set $E_{R}$. Fix a point $x_{0}\in C$ and denote $f^{-1}\left(f\left(x_{0}\right)\right)≕\left\{x_{0},x_{1},\text{…},x_{k-1}\right\}$. By Lemma 4.1(e) we have $f^{-1}\left(f\left(x_{0}\right)\right)\subset C$. As C is path‐connected by definition, we may now take an injective path α:[0,1]→C with $\alpha \left(0\right)=x_{0}$ and $\alpha \left(1\right)=x_{1}$. The image of this path, $\beta ≔f\circ \alpha :\left[0,1\right]\to f\left(E_{R}\right)$, is a loop based at $y_{0}≔f\left(x_{0}\right)$. If β was zero‐homotopic in $f(E_{R})$, we could lift the homotopy with the covering map ${f|}_{E_{R}}:E_{R}\to f\left(E_{R}\right)$ into a homotopy in $E_{R}$ contracting the path α to a point without changing the endpoints of the path, see [14, Proposition *1.30*]. This is not possible when k⩾2, and so we must have [β]≠0 in $\pi_{1}\left(f\left(E_{R}\right),y_{0}\right)$. Likewise, since f is a proper map, its restriction to $B\setminus f^{-1}\left(f\left(B_{f}\right)\right)$ is also a covering map and so β is not zero‐homotopic also in $f\left(B\right)\setminus K\subset f\left(B\right)\setminus f\left(B_{f}\right)$.

Next we construct a loop $\gamma :\left[0,m\right]\to E_{R}$, see again Figure *1*. We set first $\gamma_{1}=\alpha$. Then, when $\gamma_{k}:\left[0,k\right]\to E_{R}$ has been defined and if $\gamma_{k}\left(0\right)\neq \gamma_{k}\left(1\right)$, we define $\gamma_{k+1}$ by lifting the path β from the point $\gamma_{k}\left(1\right)$ with Lemma 2.2 and concatenating that lift to $\gamma_{k}$. Since the covering map is a local homeomorphism, this procedure is well defined and since $f^{-1}\left(f\left(x_{0}\right)\right)$ is finite, it terminates after at most k steps.

But now we note that

$$\begin{array}{c} \left|\gamma \right|\subset E_{R}\subset B\setminus {\bar{B}}_{R}\subset E_{s}, \end{array}$$

and so by the assumption n⩾3 the loop γ can be contracted to a point in the spherical shell $B\setminus {\bar{B}}_{R}$ and thus in $E_{s}$. This contracting homotopy can then be pushed with the covering map ${f|}_{E_{s}}$ into $f(E_{s})$, and so we see that

$$\begin{array}{c} 0=\left[f\circ \gamma \right]={\left[\beta \right]}^{m}, \end{array}$$

and so we see that [β] is a non‐trivial torsion element in $\pi_{1}\left(E_{s},y_{0}\right)$. Since, as noted before, [β]≠0 also in $\pi_{1}\left(f\left(B\right)\setminus K\right)$ and clearly ${\left[\beta \right]}^{m}=0$ in $\pi_{1}\left(f\left(B\right)\setminus K\right)$, we see that [β] is also a non‐trivial torsion element in f(B)∖K. This is a contradiction and so the original claim holds.□

Our proof in dimension three relies on the following result of Papakyriakopoulos, see [26, Corollary 31.8].

Proposition 4.4 Let $\Omega \subset R^{3}$ be a domain. Then $\pi_{1}\left(\Omega \right)$ is torsion‐free.

By Proposition 4.4 any domain in $R^{3}$ has torsion‐free fundamental group, in particular, it has torsion‐free fundamental group at infinity. This yields the proof of Theorem 1.1.

Proof of Theorem 1.1 Let $f:B^{3}\to f\left(B^{3}\right)\subset R^{3}$ be a proper branched covering and denote $Y≔f(B^{3})$. By Proposition 4.4 we know that for any compact set K⊂Y, the fundamental group of Y∖K is torsion‐free. Thus Y has torsion‐free fundamental group at infinity, and the claim follows from Proposition 4.3.□

Remark 4.5 As mentioned in the introduction *(*see Proposition 1.3) the assumptions on the spaces can be slightly relaxed. However, some structure is required. For example, if the target of f is not assumed to be a manifold, we may take the the universal covering map $p:S^{2}\to P^{2}$ onto the projective plane $P^{2}$ and let f be the *cone map* *(*see, e.g., [24] for the terminology*)*

$$\begin{array}{c} \mathrm{cone}\left(p\right):\mathrm{cone}\left(S^{2}\right)=\bar{B^{3}}\to \mathrm{cone}\left(P^{2}\right). \end{array}$$

The mapping f restricted to the open ball $B^{3}$ is a proper branched covering onto a space which is an open *3*‐manifold outside one singular point. Furthermore $B_{f}=\left\{0\right\}$, so in particular the branch is non‐empty but compact. Similar examples appear from universal covers of homology spheres. Thus we must assume that the image of f is a manifold. We do remark that we do not know if the Vuorinen question holds for mappings $f:B^{3}\to N$ where N is a 3‐manifold not necessarily embeddable into $R^{3}$.

Remark 4.6 In [3] it was proved that there exist *essentially proper* † branched coverings $f:B^{3}\to R^{3}$ with $B_{f}=\emptyset$ that are not homeomorphisms. In the same paper it is claimed that one can obtain a proper branched covering by restricting the above mapping f to a ball $B_{r}$ with radius r<1 arbitrarily close to 1. However, no detailed argument is provided and the claim is not true for essentially proper branched coverings in general. It turns out that every open continuous map $g:B^{n}\to R^{3}$ is essentially proper. This can be seen as follows: Let $K\subset g(B^{n})$ be an arbitrary compact set. Since g is open, the sets ${\left\{g\left(B_{r}\right)\right\}}_{r\in (0,1)}$ form an open covering of K. Thus, by compactness we find a ball $B_{r_{0}}$ such that ${\bar{B}}_{r_{0}}\cap g^{-1}\left(K\right)$ is compact and $K=g({\bar{B}}_{r_{0}}\cap g^{-1}\left(K\right))$. Especially, the mapping mentioned in (1.1) is essentially proper but cannot be made into a proper mapping by restricting it to a slightly smaller box. Theorem 1.2 shows that a counterexample to Vuorinen's question cannot have an empty branch set in any dimension.

## JOURNAL INFORMATION

The *Bulletin of the London Mathematical Society* is wholly owned and managed by the London Mathematical Society, a not‐for‐profit Charity registered with the UK Charity Commission. All surplus income from its publishing programme is used to support mathematicians and mathematics research in the form of research grants, conference grants, prizes, initiatives for early career researchers and the promotion of mathematics.

## Footnotes

† We remark that the path‐lifting method in [29] assumes that the map is sense‐preserving. This is merely a notational convention as a continuous, open, and discrete mapping is always either sense‐preserving or sense‐reversing, see [29, p. 18].

† A mapping $f:B^{n}\to f\left(B^{n}\right)$ is called *essentially proper* if for every compact set $C\subset f(B^{n})$ there is a compact subset $C^{\prime }\subset B^{n}$ such that $f(C^{\prime })=C$, see [3, p. 761].
